# Influence of Standard Laboratory Procedures on Measures of Erythrocyte Damage

**DOI:** 10.3389/fphys.2017.00731

**Published:** 2017-09-29

**Authors:** Lena Wiegmann, Diane A. de Zélicourt, Oliver Speer, Alissa Muller, Jeroen S. Goede, Burkhardt Seifert, Vartan Kurtcuoglu

**Affiliations:** ^1^The Interface Group, Institute of Physiology, University of Zurich, Zurich, Switzerland; ^2^National Center of Competence in Research, Kidney.CH, Zurich, Switzerland; ^3^Division of Haematology, University Children's Hospital Zurich, Zurich, Switzerland; ^4^Department of Health Sciences and Technology, ETH Zurich, Zurich, Switzerland; ^5^Department of Haematology, Kantonsspital Winterthur, Winterthur, Switzerland; ^6^Zurich Center for Integrative Human Physiology, University of Zurich, Zurich, Switzerland; ^7^Department of Biostatistics, Epidemiology, Biostatistics and Prevention Institute, University of Zurich, Zurich, Switzerland; ^8^Neuroscience Center Zurich, University of Zurich, Zurich, Switzerland

**Keywords:** red blood cells, erythrocytes, centrifugation, vortexing, pipetting, free hemoglobin, ektacytometry

## Abstract

The ability to characterize the mechanical properties of erythrocytes is important in clinical and research contexts: to diagnose and monitor hematologic disorders, as well as to optimize the design of cardiovascular implants and blood circulating devices with respect to blood damage. However, investigation of red blood cell (RBC) properties generally involves preparatory and processing steps. Even though these impose mechanical stresses on cells, little is known about their impact on the final measurement results. In this study, we investigated the effect of centrifuging, vortexing, pipetting, and high pressures on several markers of mechanical blood damage and RBC membrane properties. Using human venous blood, we analyzed erythrocyte damage by measuring free hemoglobin, phosphatidylserine exposure by flow cytometry, RBC deformability by ektacytometry and the parameters of a complete blood count. We observed increased levels of free hemoglobin for all tested procedures. The release of hemoglobin into plasma depended significantly on the level of stress. Elevated pressures and centrifuging also altered mean cell volume (MCV) and mean corpuscular hemoglobin (MCH), suggesting changes in erythrocyte population, and membrane properties. Our results show that the effects of blood handling can significantly influence erythrocyte damage metrics. Careful quantification of this influence as well as other unwanted secondary effects should thus be included in experimental protocols and accounted for in clinical laboratories.

## Introduction

Erythrocytes, or red blood cells (RBCs), constitute the majority of blood cellular components and are responsible for the vital transport of oxygen and carbon dioxide throughout the body. The mechanical properties and physical integrity of the erythrocyte plasma membrane are central to this function, allowing RBCs to undergo considerable deformations and travel through the smallest capillaries. In converse, impaired RBC membrane properties and deformability yield severe pathological phenotypes, including sickle cell anemia, spherocytosis, stomatocytosis, and elliptocytosis. Beyond hereditary diseases, blood damage is also often an acquired condition due to cardiovascular implants such as artificial heart valves or ventricular assist devices (Shapira et al., 2009; Kirklin et al., 2015). The ability to characterize RBC mechanical properties is therefore important in both clinical and research contexts: firstly, to diagnose and monitor hematological disorders and, secondly, to understand hemolysis pathways and optimize the design of implantable or extracorporeal devices in order to minimize the induced blood damage.

Mechanical forces acting on RBCs range from those found physiologically in the cardiovascular system to pathophysiological stresses present in implants or during (inappropriate) handling. The range of the cells' reaction naturally also varies from deformation to changes in volume to cell rupture. The essential role in volume homeostasis of Piezo1, a mechanically activated cation channel in the RBC membrane, was recently shown (Cahalan et al., 2015). Activation of Piezo1 due to mechanical loading leads to Ca^2+^ influx, which in turn triggers the dehydration of the cells. This mechanism of volume reduction in response to stress could improve the RBCs' ability to travel through the smallest capillaries (Cahalan et al., 2015) and was also hypothesized to promote oxygen/CO_2_ exchange in the periphery, which was observed following mechanical stimulation of RBCs (Rao et al., 2009). If the stresses acting on the cell exceed its loading capacity, pore formation (Zhao et al., 2006) or complete membrane rupture (Rand, 1964) occurs, and all cytosolic content including hemoglobin is released into the plasma.

In clinical laboratories, several parameters are carefully and specifically analyzed to judge a patient's hematological status. Standardized operating procedures, careful selection of controls and participation in round robin tests ensure a high quality and reproducibility of the results (Gunter et al., 1996; Lippi et al., 2006; Plebani, 2007). At the same time, protocols involve several preparation and intermediate steps, such as pipetting, mixing, and centrifuging, which impose mechanical stresses on the cells. Little is known about their influence on the final laboratory results.

This similarly holds true for *in vitro* experiments for research purposes and the systematic investigation of shear stress effects on cell integrity (Zhao et al., 2006; Quinn et al., 2011). To reproduce mechanical stresses in artificial organs such as ventricular assist devices or across artificial heart valves, researchers often rely on *in vitro* systems such as viscometers or microchannels (Leverett et al., 1972; Sutton et al., 1997; Paul et al., 2003; Korin et al., 2007). Yet, irrespective of proper control of the shear stress levels within the actual experimental apparatus, these experiments again include handling steps for the preparation of cells, for their placement in the apparatus and for preparation of samples for subsequent analysis. Secondary experimental effects, inherent to the geometric configuration and setup of the apparatus, may also influence the measurement end-points. One such example is the occurrence of high pressures required to drive blood through microchannels. A thorough understanding of the contribution of these different forces to the measurement endpoints is therefore of critical importance for the interpretation of the results and optimization of the laboratory procedures. Still, to date, little literature is available addressing this.

Damage due to centrifugal forces has been demonstrated on different cell lines, but not on erythrocytes (Katkov and Mazur, 1998; Ferraro et al., 2011). Similarly, the effect of pipetting and elevated pressures was studied on other cell types (Kay et al., 2004; Heng et al., 2007). Various mixing methods were compared (Bai et al., 2012), identifying vortexing as the most stress intensive method. However, the induced cell damage was not quantified. For RBCs, the influence of standard laboratory procedures on hemolysis has mostly been studied in the context of transfusion medicine. Delay between collection and separation, large variation in centrifuging speeds, rapid anticoagulation, as well as shaking and mixing were found to yield increased levels of hemolysis in blood bags (Sowemimo-Coker, 2002). Along the lines of testing handling artifacts on RBCs, the effect of different modes of transportation, reflected in variations in anticoagulation and temperature in simulated shipment conditions, was investigated in Makhro et al. (2016).

While these studies clearly indicate that potentially any handling associated with large forces may be detrimental to cells, it remains unclear to what extent and in what fashion RBCs will incur damage. Next to hemolysis, mechanical stress-induced changes to the plasma membrane deserve attention. Here we address this knowledge gap by investigating the effect of centrifuging, vortexing, pipetting, and high pressures on several markers of mechanical blood damage and erythrocyte membrane properties.

## Materials and methods

### Subjects

We used venous whole blood of 22 healthy volunteers. Blood was collected with a syringe from a venous catheter (20G) in an antecubital vein. It was anticoagulated either with lithium heparin or ethylenediaminetetraacetic acid (EDTA) depending on the subsequent analysis. The study was approved by the Ethics Commission of the Canton of Zurich, and conformed to the Declaration of Helsinki. Subjects gave written informed consent to participation. The collected samples were randomly assigned to experimental setups and analyses and each donor's blood was used for multiple experiments. Experiments were started immediately after drawing blood and performed at room temperature. The experimental order was randomized and the last experiment was always completed within a maximum of 2 h of blood draw. Blood awaiting experimental testing was kept at 4°C to minimize changes in hemorheological properties (Baskurt et al., 2009). The analysis procedures as described in section Analyses were initiated right after the end of the experiments and completed within a maximum of 6 h. The experimental and analysis workflows are explained in the next sections.

### Experimental setups

**Figure 1:**
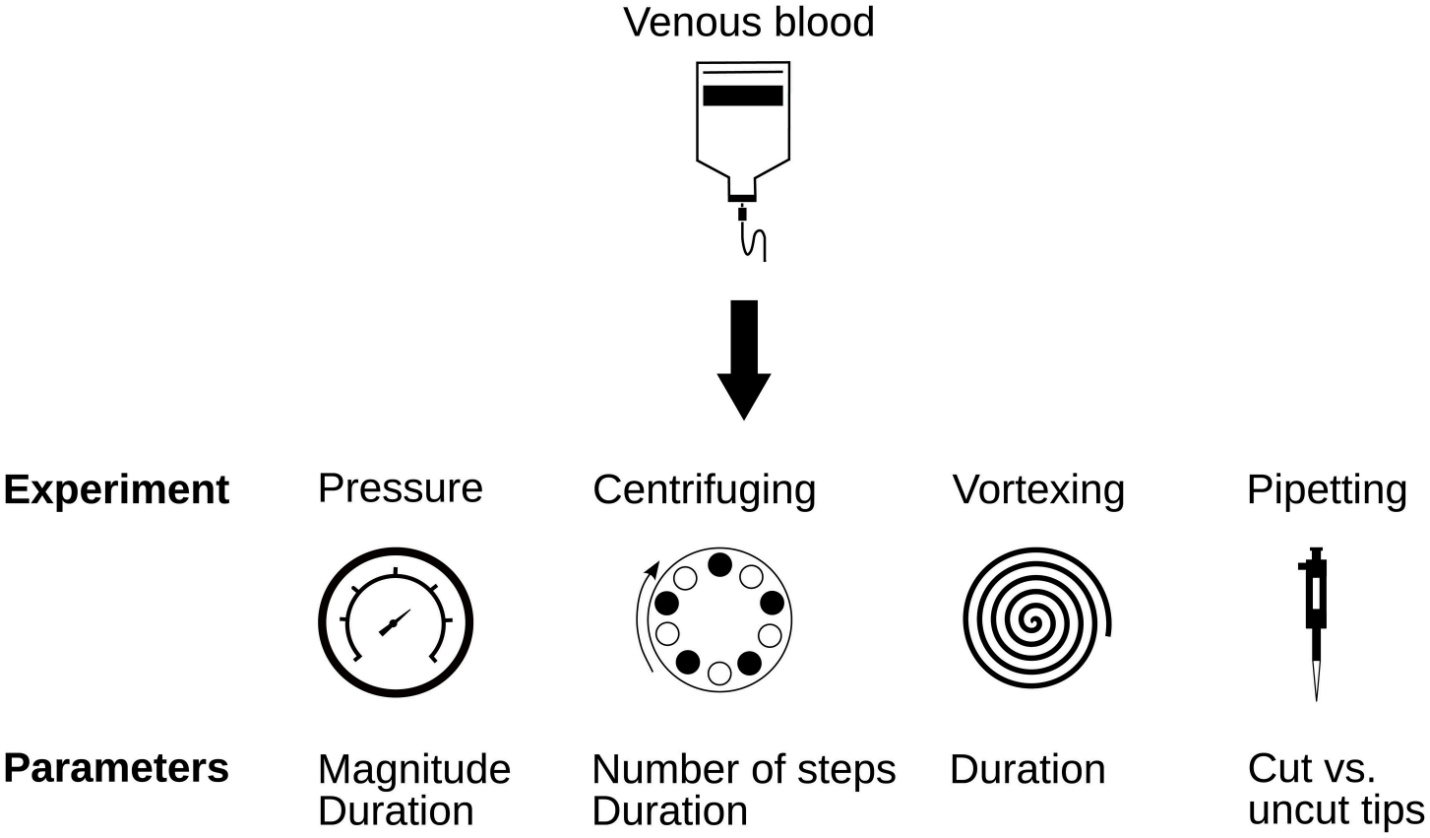
Overview over the experimental setups presented in this study.

Figure 1 provides an overview of the experimental setups presented in this study. We investigated the effect of pressure, centrifuging, vortexing, and pipetting, and for each one of these four methods tested a range of setup parameters such as stressor magnitude or exposure time. Centrifuging and pipetting are routinely used both in hematology and research laboratories. Although, vortexing is usually avoided in RBC handling, it was nevertheless included here to explore the upper bound of applied stresses in a laboratory context. Finally, repeated exposure to pressure plateaus was motivated by the conditions typically experienced by RBCs in experimental microchannel setups. Detailed descriptions of the different experimental conditions are provided in the following subsections.

#### High pressure

*In-vitro* microchannel setups typically involve repeated cell exposure to high pressures, required to drive the cell suspension through channels and constrictions. To assess the effect of these repeated pressure exposures independently of other forces, notably independently of shear stresses, the samples were exposed to pressure sequences in custom-built pressure chambers driven by compressed nitrogen. A pressure sequence consisted of square pressure waveforms including 10 repeated cycles of high and low pressures. High pressure plateaus (P_high_) were maintained for a duration T_high_, where P_high_ was set to either 3, 5, or 7 bars and T_high_ to 1 or 30 s for short or long exposures, respectively. T_high_ and P_high_ remained constant within a given experimental sequence. In all sequences, samples were allowed to recover for 30 s at low (ambient) pressure between two consecutive high pressure plateaus. Pressure relative to the atmosphere was monitored with a pressure sensor (PBT-RB010SG1SSNAMA0Z, Sick AG, Waldkirch, Germany). The number of tested samples is indicated in the respective figure caption in the Results section. Controls for this experiment were kept at room temperature in petri dishes (Fisher Scientific, Waltham MA, USA) as used in the pressure chambers for 5 min (short exposure experiment) respectively 10 min (long exposure experiment).

#### Centrifugation

All centrifugation experiments were performed in the same benchtop centrifuge (Eppendorf Centrifuge 5415D, Eppendorf AG, Hamburg, Germany) at 900 g, a centrifuging speed that was chosen according to a diagnostic standard operation procedure at the erythrocyte laboratory, University Children's Hospital, Zurich. Samples were centrifuged for 5 or 10 consecutive minutes; 1, 2, or 4 times in a row. Energic mixing is required to resuspend the pellet before analysis or the next centrifugation cycle. In these experiments, the samples were vortexed for 2 s after each centrifugation. Controls were kept at room temperature in Eppendorf tubes as used in the experiments and vortexed for 2 s before analysis. The number of tested samples is indicated in the figure caption in the Results section. Per design, comparison of the controls vs. single centrifugation elucidates damage induced by centrifugation only, while comparison against samples centrifuged two to four times, demonstrates the damage induced by repeated centrifuging and mixing/vortexing cycles (see also section Discussion for in depth discussion).

#### Vortexing

Vortexing was included as representative of extreme stressors on RBCs. Samples were placed into 1.5 ml Eppendorf tubes and vortexed once for 20 or 40 s in a standard laboratory vortexer (Genie, VWR International, Rednor, USA). The number of tested samples is indicated in the figure caption in the Results section. Controls were kept at room temperature in the same Eppendorf tubes without vortexing.

#### Pipetting

To test whether cutting the front end of pipet tips makes pipetting less harmful for RBCs, we compared samples that were pipetted in and out 10 times in a row with and without cut tip to controls. The repeated exposure was carried out to amplify possible effects on the cells. Pipet tips used were standard 1,000 μl tips (Tip One, Starlab, Milton Keynes, United Kingdom). Cut tips were cut 7 mm above the tip end. The number of tested samples is indicated in the figure caption in the Results section. Controls were kept at room temperature in the same Eppendorf tubes as used in the experiments.

Naturally, all experiments involved one pipetting step before and after the experiment. It was pursued as gently as possible to keep forces on the cells as low as possible. As a reference, the pipetting rate used in the pipetting experiments was 639 μl/s (51.1 ± 1.1 s for 10 consecutive cycles of pipetting 1,000 μl), compared to a rate of 392 μl/s for the gentle pipetting. Since these gentle pipetting steps are also applied on every control sample, the measured differences are not affected.

### Analyses

**Figure 2:**
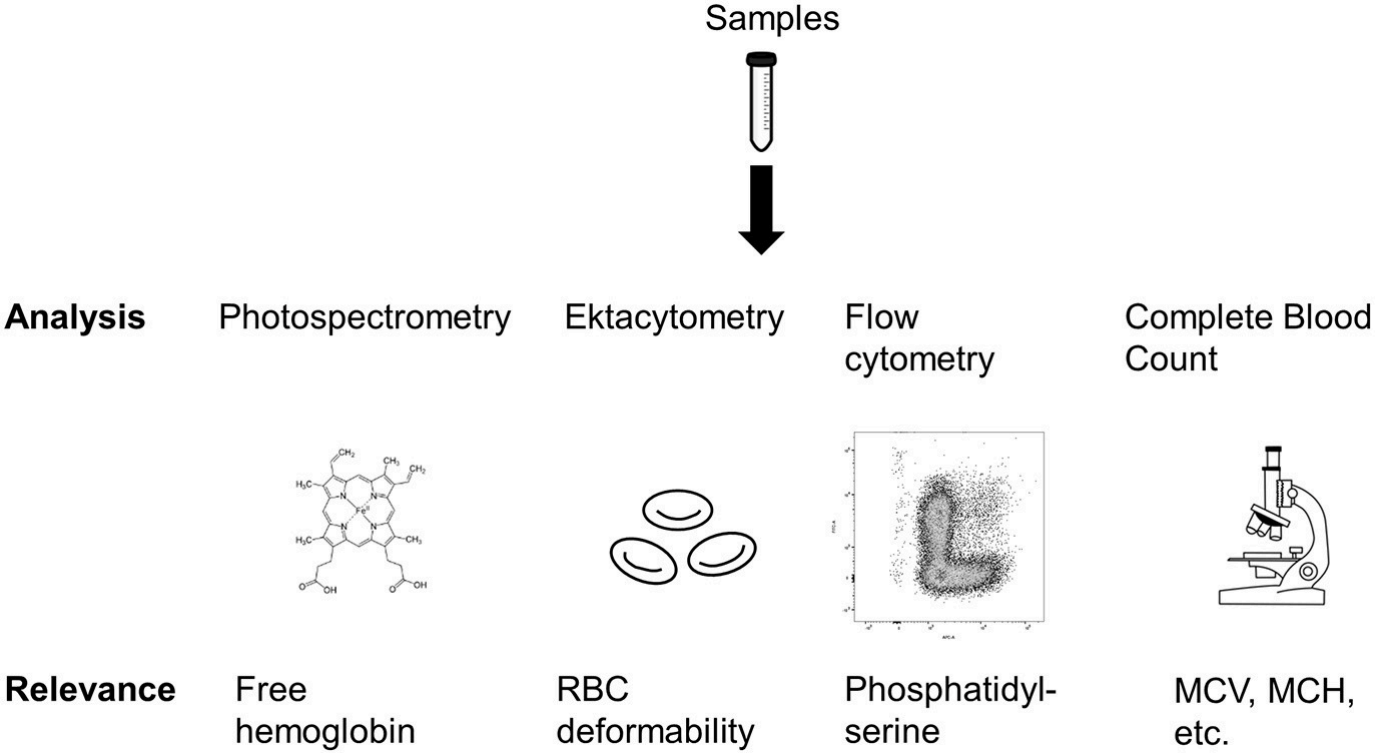
Overview of the analyses performed in this study. MCV, Mean cell volume; MCH, Mean corpuscular hemoglobin. Sources: Tube: https://pixabay.com/en/tube-conical-test-measured-305147/; Heme: https://commons.wikimedia.org/wiki/File:Heme_b.svg; Microscope: https://pixabay.com/en/microscope-lab-chemistry-science-2223268/.

Figure 2 provides an overview of the analyses conducted in this study. We analyzed multiple parameters indicative for mechanical blood damage and RBC membrane properties.

#### Free hemoglobin

The samples were anticoagulated using lithium heparin. To avoid additional mechanical stresses, plasma separation was not achieved via centrifuging but via sedimentation for 180 min. The sedimentation period was started immediately after finishing the experiments. After separation, the plasma was pipetted gently and frozen at −20°C for later analysis.

Free hemoglobin (Hb) in plasma was measured using photospectrometry at 415, 450, and 700 nm and the final Hb concentration was calculated according to (Fairbanks et al., 1992):

$$C_{Hb}[\text{mg/ml}]=1.55\cdot A_{415}-1.3\cdot A_{450}-1.24\cdot A_{700}$$

The first term represents the known absorption coefficient of hemoglobin, while the second and third term correct for absorption by bilirubin and turbidity. Note that this formulation can yield negative values, which clinically are cut-off and represented by a concentration of 0 mg/ml free hemoglobin. In this study, we kept the original values (whether positive or negative) in order to maintain a valid distribution for subsequent statistical analysis. Triplets of each sample were measured. The reported value corresponds to the arithmetic mean of the three measurements.

#### Ektacytometry

Ektacytometry is currently the most widely used technique for the measurement of RBC deformability (Baskurt et al., 2009). Clinically, it is applied for the diagnosis of several erythrocyte membranopathies, such as hereditary spherocytosis, stomatocytosis, and ellipsocytosis. Here, we used this method to detect changes in the general deformation behavior of the cells. In an ektacytometry measurement, EDTA anticoagulated whole blood is mixed with viscous solutions of different osmolalities, and the elongation index (EI) of the RBCs under constant shear is measured using laser diffraction. The minimal osmolality with measurable EI (Omin) corresponds to the osmotic fragility measured in other applications. All measurements were performed in a Lorrca Maxsis Osmoscan (RR Mechatronics, Hoorn, The Netherlands). Blood samples were analyzed according to the standard protocol for this machine, which involves mixing of the sample in iso-osmolar polyvinylpyrrolidone solution (RR mechatronics) before analysis.

#### Phosphatidylserine expression

Phosphatidylserine is a membrane phospholipid and its externalization a marker for macrophage clearance (Boas et al., 1998). Therefore, it is also interpreted as a marker of RBC damage (Lutz and Bogdanova, 2013). We measured its expression levels using flow cytometry using a protocol similar to the one described in Kuypers et al. (1996).

##### Normal sample preparation

Blood was anticoagulated using lithium heparin. Erythrocytes were labeled using allophycocyanin (APC) anti-human CD235ab antibody staining and PS expression was measured using fluorescein isothiocyanate (FITC) labeled annexin V. All reagents were ordered from BioLegend Europe (London, United Kingdom). Cells were incubated for 20 min in complete darkness after mixing 5 μl of APC-CD235ab and FITC-annexin V with 95 μl of annexin binding buffer and 5 μl of whole blood. Four hundred microliters of buffer where then added and everything was mixed using the vortex before the solution was transferred to Falcon polystyrene tubes (Fisher Scientific, Waltham MA, USA), which were prefilled with 500 μl of the same buffer.

##### Controls

We used the following controls: an unstained tube, an APC negative control, a FITC negative control, the two Fluorescence Minus One (FMO) controls and a fully stained control with a 50/50 mixture of normal and PS-positive blood. For the APC negative control, we used a peripheral blood mononuclear cell (PBMC) solution. The unstained control was intended to detect the level of background fluorescence of the system. The FITC negative control contained phosphate buffered saline (PBS) buffer instead of annexin V binding buffer. In the FMOs and the all-in control, we used blood that was prepared to contain an increased amount of PS-positive cells. Details of these protocols are described hereafter.

##### Preparation of PS-positive blood

Blood was treated with *N*-Ethylmaleimide (NEM; Sigma-Aldrich, St. Louis, Missouri, USA), which inhibits the aminophospholipid translocase, and afterwards mixed with calcium ionophore A23817 (Sigma-Aldrich), to induce membrane lipid scrambling (Kuypers et al., 1996). After washing the cells once with PBS, 10 mM NEM buffer solution was added to resuspend the cell pellet. The solution was then incubated for 30 min, and afterwards centrifuged for 5 min at 835 g to remove the supernatant. After another washing step, the cell pellet was resuspended in 2 mM calcium buffer solution and incubated during 3 min at 37°C. Six microliters of 1 mM calcium ionophore solution (in DMSO) were then added and incubated during 1 h at room temperature. Thereafter, the solution was centrifuged for 5 min at 835 g to remove the supernatant. The cells were washed once with 2.5 mM EDTA buffer solution and three times with cell staining buffer (BioLegend Europe). The cell pellet was then resuspended in 0.3 ml PBS and used as what is described here as “PS-positive blood.”

##### Preparation of PBMCs

A solution of PBMCs was produced using Ficoll-Paque separation. Whole blood was diluted in 2–4x 2 mM EDTA buffer (pH 7.2). For the density gradient centrifugation, 5 ml of Ficoll (GE Healthcare, Little Chalfont, United Kingdom) were filled into a 15 ml tube, afterwards 7 ml of the diluted blood was added. After centrifugation at 800 g for 20 min, PBMCs were gently pipetted off, washed with PBS and the cell pellet resuspended in PBS.

##### Flow cytometry

The flow cytometric analysis was performed in a BD FACS Canto II (BD Biosciences, Franklin Lakes, New Jersey, USA). In our gating strategy, we initially gated for singlets using forward scatter height vs. width. Afterwards, we selected APC-positive cells (erythrocytes) and, finally, compared the number of FITC-positive cells across samples.

#### Complete blood count (CBC)

A routine CBC was conducted using an Advia 2120i system (Siemens Healthcare, Erlangen, Germany) in all samples that also underwent ektacytometry testing.

### Statistics

Numerical values are reported as either absolute or normalized by their respective control to account for inter-subject variability. Statistical analyses were conducted using the IBM SPSS Statistics 23 software (IBM Corporation, Armonk, USA). We performed a linear mixed model analysis with the subject as random effect. *Post-hoc* analysis was according to Tukey-HSD (Tukey, 1977). *P*-values below 0.05 are considered significant and are indicated in the respective figures.

## Results

### Pressure

**Figure 3:**
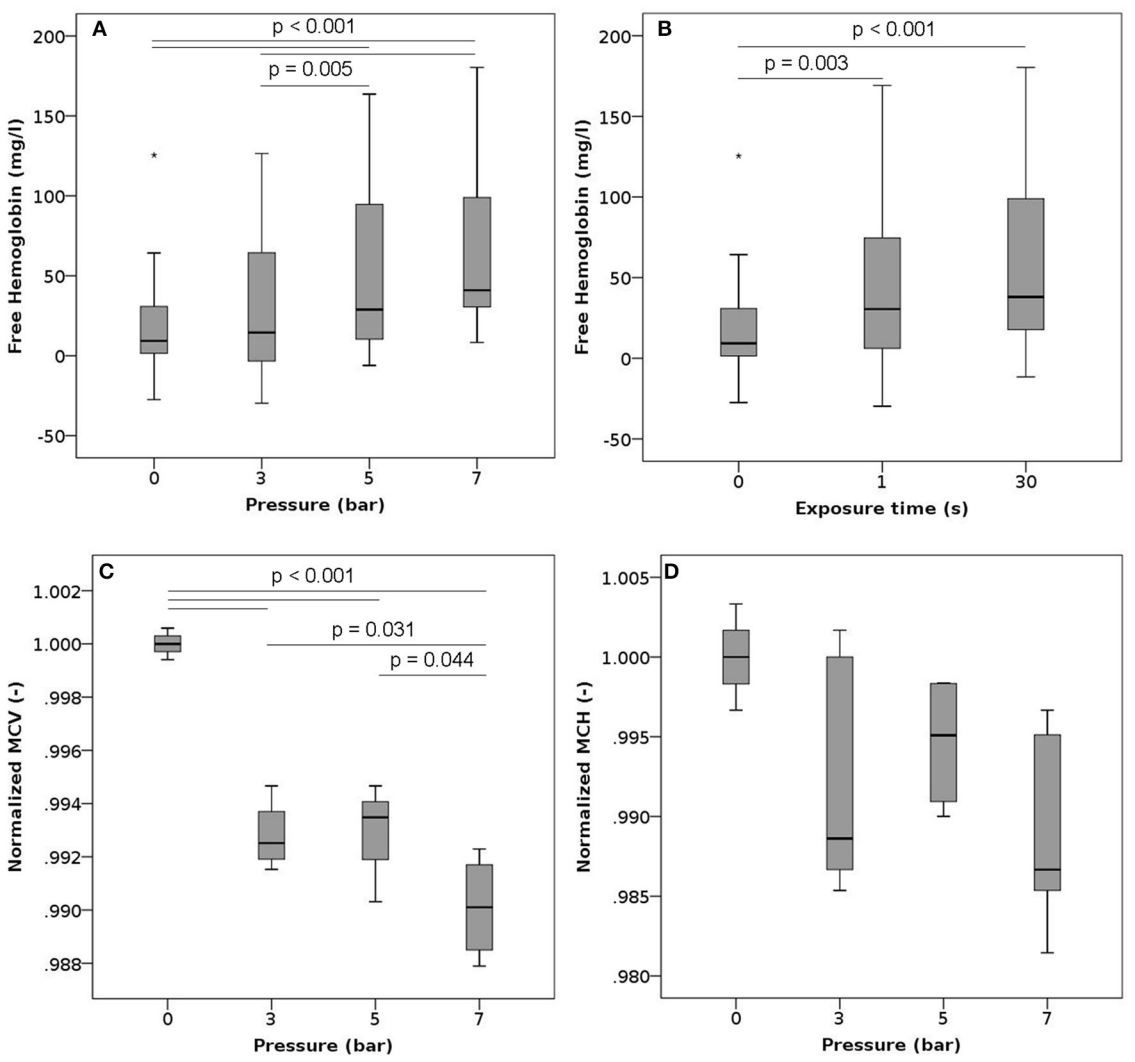
Free hemoglobin, MCV and MCH after exposure to high pressure. Blood samples were exposed to high pressures (varying between 3 and 7 bars) 10 times (single exposure duration of 1 or 30 s) with a 30 s recovery period between two consecutive exposures. **(A)** Free hemoglobin as a function of the pressure level (irrespective of the exposure time) *n* = 18, 22, 20, and 21 for high pressures of 0, 3, 5, and 7 bar, respectively. **(B)** Free hemoglobin as a function of the exposure duration (irrespective of the pressure level) *n* = 18, 33, and 30 for exposures of 0, 1, and 30 s, respectively. **(C)** Normalized mean cell volume as a function of the pressure level (exposure time 1 s). Reported values have been normalized by the mean of the corresponding control measurements. *n* = 4, 4, 3, and 4 for high pressures of 0, 3, 5, and 7 bar, respectively. **(D)** Normalized mean corpuscular hemoglobin as a function of the pressure level (exposure time 1 s). Reported values have been normalized by the mean of the corresponding control measurements. *n* = 4, 4, 3, and 4 for high pressures of 0, 3, 5, and 7 bar, respectively.

We exposed the samples to a series of high pressure peaks, lasting 1 or 30 s each. Exposure to high pressures significantly increased plasma free hemoglobin (Figures 3A,B). The higher the pressure level, the more hemoglobin was released from the RBCs. While the magnitude of the pressure had a significant effect on free Hb levels, there was no difference between the two exposure times suggesting that the duration of the exposure did not play a major role. Increasing pressures were also associated with a significant decrease in mean cell volume (MCV, Figure 3C). The mean corpuscular hemoglobin (MCH, Figure 3D) decreased with increasing pressure, but this decrease was not statistically significant, while the MCH concentration (MCHC, Figure S1) stayed constant. Neither the ektacytometric measurement nor the phosphatidylserine expression showed any differences for samples exposed to elevated levels of pressure compared to controls (Figures S4, S8A).

### Centrifugation

**Figure 4:**
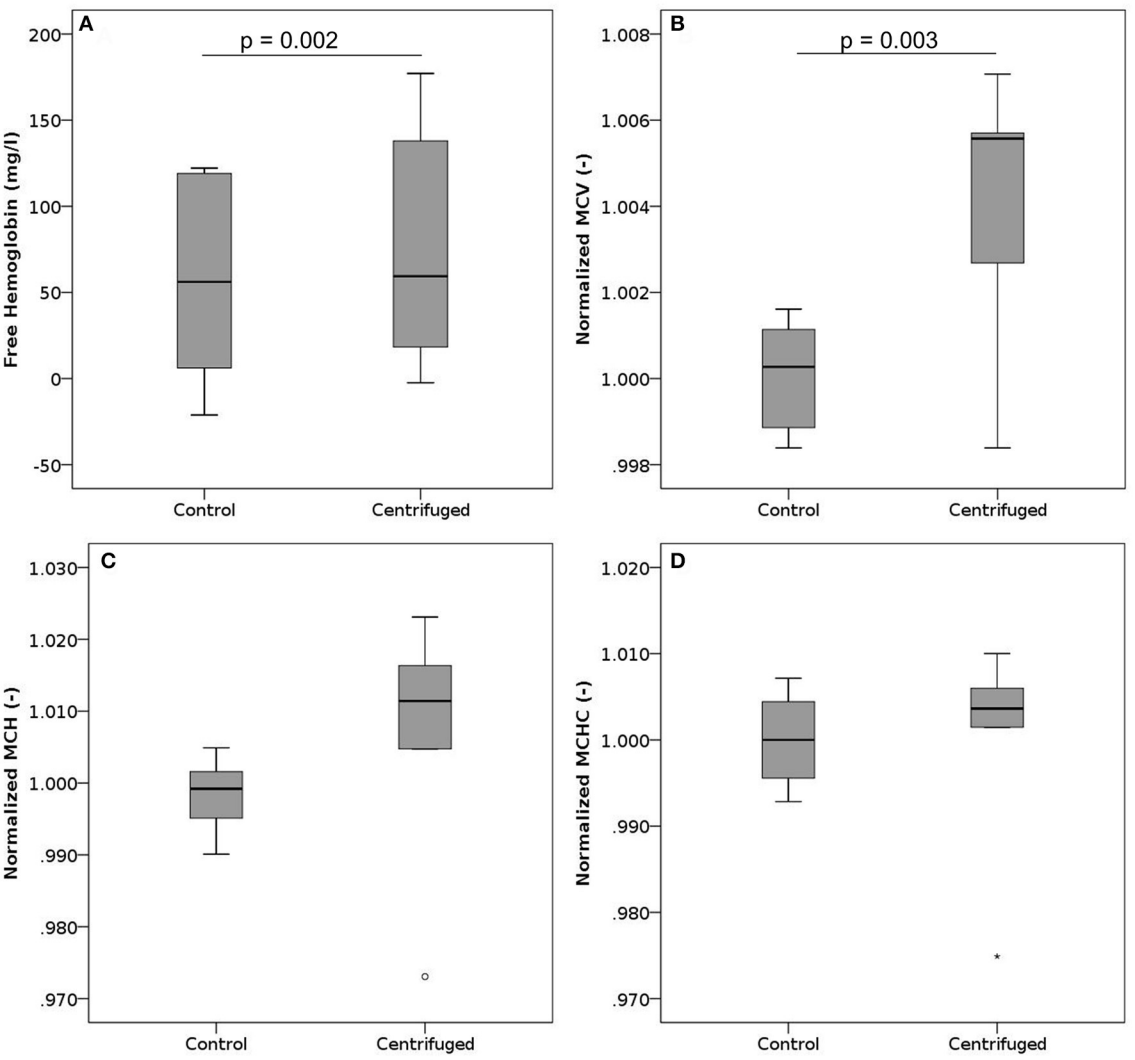
Free hemoglobin, MCV, MCH, and MCHC after centrifugation. Blood samples where centrifuged at 900 g. **(A)** Free hemoglobin after centrifugation for 5 or 10 min. The black line indicates the median, while the mean values are 61 mg/l and 79 mg/l for controls and centrifuged samples, respectively. *n* per group = 12. **(B)** Normalized mean cell volume after centrifugation for 5 min. Reported values have been normalized by the mean of the corresponding control measurements. *n* per group = 6. **(C)** Normalized mean corpuscular hemoglobin after centrifugation for 5 min. Reported values have been normalized by the mean of the corresponding control measurements. *n* per group = 6. **(D)** Normalized mean corpuscular hemoglobin concentration after centrifugation for 5 min. Reported values have been normalized by the mean of the corresponding control measurements. *n* per group = 6.

In these experiments, we centrifuged samples at 900 g for either 5 or 10 min. Sample centrifugation increased the plasma free hemoglobin from a mean baseline value of 61 mg/l up to 79 mg/l (Figure 4A). This behavior was observed for samples centrifuged for 5 min as well as for those centrifuged for 10 min. Overall, we observed higher absolute free Hb values for samples centrifuged for 5 min.

The MCV increased slightly with centrifugation (Figure 4B), while MCH (Figure 4C) and MCHC (Figure 4D) did not change significantly, though showing a trend to increase. Neither phosphatidylserine expression nor parameters measured in ektacytometry were influenced by centrifugation (Figures S5, S8B).

To assess the influence of more than one centrifugation step, the samples had to be resuspended in between centrifugations by a short vortexing step. Consequently, we measured a combined effect of centrifuging and vortexing for these samples. Free Hb increased with an increasing number of centrifugation and resuspension steps (Figure S9A), while maximum deformability as measured by ektacytometry, EI_max_, slightly decreased (Figure S9D).

### Vortexing

**Figure 5:**
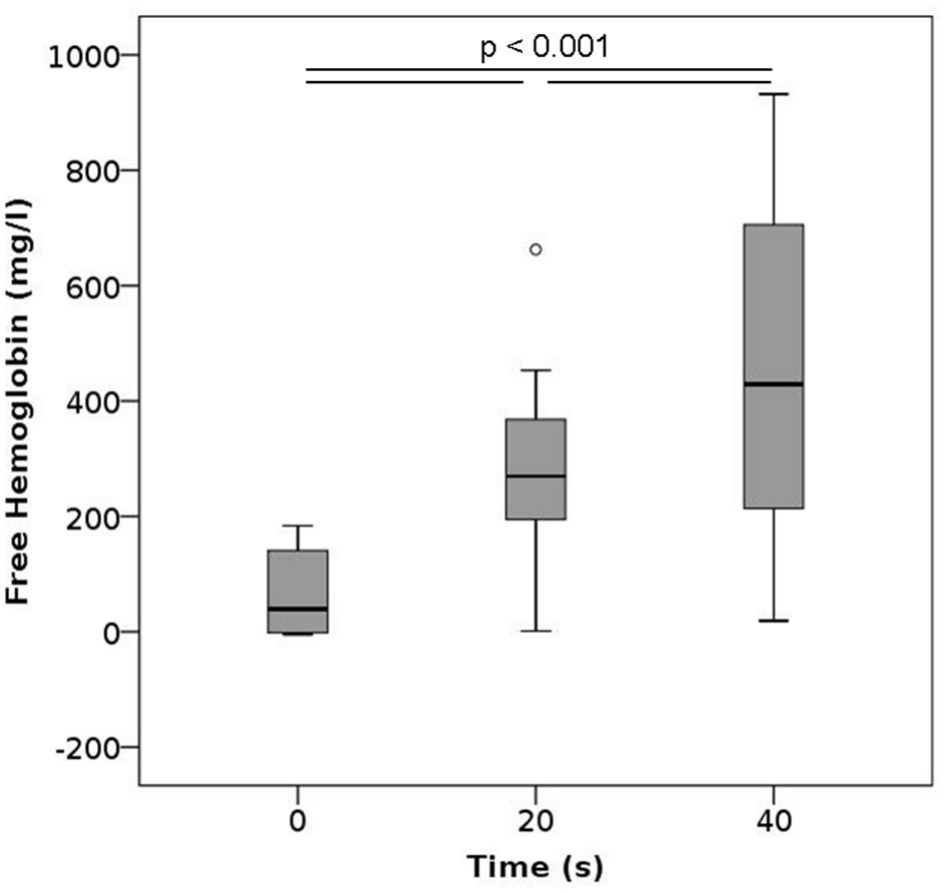
Free hemoglobin after vortexing as a function of the vortexing time. *n* per group = 12.

In these experiments, blood samples were vortexed for either 20 or 40 s. Such vortexing led to significant increase in plasma free hemoglobin (Figure 5), resulting in the highest levels of free hemoglobin in plasma observed within this study. This increase in released Hb was strongly dependent on the duration of vortexing. None of the characteristics measured within the complete blood count, ektacytometry, and measurement of PS-expression showed any significant changes after vortexing (Figures S2, S6, S8B).

### Pipetting

**Figure 6:**
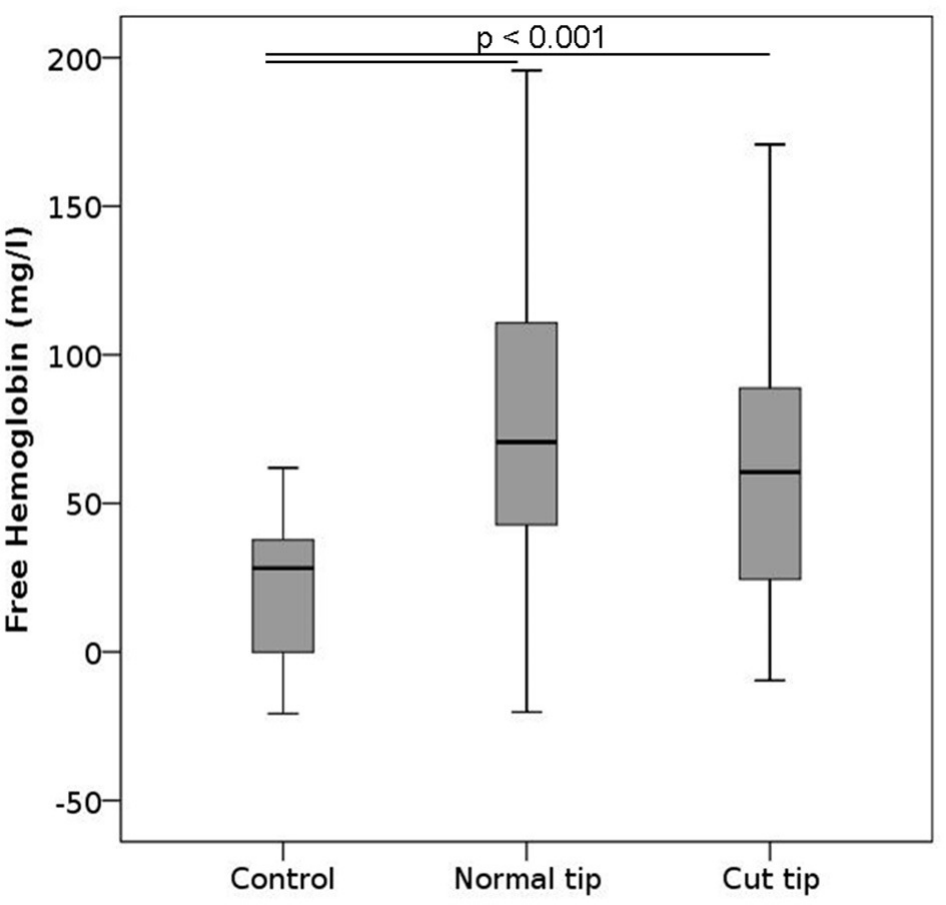
Free hemoglobin after pipetting. Blood samples were primed and relieved from the pipet 10 consecutive times. The pipet tip was either normal or cut 7 mm away from the leading edge. *n* per group = 12.

To test the effects of pipetting and strategies proposed to potentially moderate them, we pipetted the samples 10 times in a row using either normal or cut pipet tips. Pipetting induced a statistically significant increase in free hemoglobin compared to controls irrespective of the tip used (Figure 6), and there was no difference between samples that were pipetted with normal or cut tips. No changes were noted in the ektacytometry curves, phosphatidylserine expression or in any of the parameters measured within the CBC (Figures S3, 7, S8C).

## Discussion

While the connection between mechanical forces and hemolysis is well-acknowledged, it has mostly been considered in research applications, for example to investigate shear stress-induced hemolysis in cardiovascular implants. However, RBC exposure to supra-physiological forces does not only occur in implants, but also during blood handling. Here we have investigated hemolytic and other stress-induced changes to RBC caused by exposure to high pressures, centrifuging, vortexing, and pipetting, thereby surveying the main mechanical stressors in laboratories and experimental blood handling.

Our results show that high pressures, centrifuging, vortexing, and pipetting all yield increased levels of free hemoglobin, implying that the forces associated with these methods already translate into hemolytic damage. Among these methods, vortexing whole blood samples led to the highest increase in plasma free hemoglobin, with values reaching a mean of 260 and 400 mg/l after vortexing for 20 and 40 s, respectively. Similar findings have been made in orbital shakers, wherein lysis occurred after some time, possibly due to prolonged fatigue (Zhang et al., 1995). Vortexing as well as shaking impose shear stresses on the cells, which RBCs are generally vulnerable to Leverett et al. (1972). Numerical simulations of the flow fields induced by rotators, orbital shakers, magnetic stirrers, and vortex mixers identified vortexing as the method that induced the largest shear stresses within the samples (Bai et al., 2012). While vortexing RBCs is generally avoided in hematology laboratories, we investigated it here as a representative of extreme stresses on the cells. As evidenced by our results, these stresses are clearly detrimental. Since we did not observe an effect on MCV or MCH, the release of hemoglobin induced by vortexing seems to be governed by complete lysis of the cells, irrespective of their size. Even though vortexing durations of 20 or 40 s are long in an experimental context, these results clearly show that vortexing *per se* is harmful to RBCs and should be avoided not only in clinical laboratories, but also in research applications.

Pipetting also induces shear forces on the cells, the magnitude of which increases as the pipette tip diameter decreases. Using large pipettes and cutting the foremost edge of the pipette tip to increase the tip diameter had thus been suggested as a means to reduce the stress on cells and mechanical damage (Exadactylos et al., 2003; Miao and Jiang, 2007). Investigating the effect of repetitive pipetting with standard or cut tips, our results confirmed that pipetting increases free hemoglobin compared to controls, but did not reveal any significant differences between the types of pipette tip used. The suction area of the cut tips was a factor of 14 larger than that of the regular tips, which may not have had a strong enough effect to be detected with our sample size. Alternatively, it is also possible that even though the shear stresses were significantly reduced in the cut pipet tips, the effect on erythrocyte lysis was counterbalanced by the creation of sharper edges. The latter option would point toward the need to round the edges after cutting. However, we do not have enough evidence to favor one interpretation over the other. We thus conclude that pipette tip sectioning does not appear to be effective in reducing pipetting induced hemolysis and may therefore not be a useful addition to the analysis workflow.

In the pressure experiments, plasma hemoglobin increased with higher pressure plateaus, but not with longer exposure times. The recovery pressure and time over which the pressure was varied were constant throughout the experimental conditions. Accordingly, the pressure gradient increased with increasing high pressure levels. Independence from the exposure time may thereby point to the temporal pressure gradients, more than to the absolute pressure values, as the predominant factor for the observed increase in plasma hemoglobin. This hypothesis is in accordance with previous findings in other cell lines. Pressure gradient, and not exposure duration, has been put forth as a primary determinant of epithelial cell damage (Kay et al., 2004). The fact that RBCs might be sensitive to pressure gradients thus warrants particular attention in research settings: For example, if high pressures are required to drive blood through a microchannel, then the rate of pressure changes along the channel should be considered in addition to the shear stresses experienced by the cells in the test section.

Our experiments revealed lysis and changes of mechanical properties after centrifuging RBCs, even though the applied centrifugal force (900 g) was at the lower end of the spectrum of commonly used forces in laboratories. This is especially noteworthy given that centrifugation is very commonly performed both in laboratory practice as well as in research experiments in preparatory steps. Between two consecutive centrifuging steps, cells were resuspended with a short vortex period, which is likely to have increased blood damage. Consequently, the results for multiple centrifugation steps reflect a combined effect of centrifuging and resuspension (Figure S9). However, since we already observed a statistically significant increase in free hemoglobin between controls and one centrifugation step (mean values of 61 mg/l vs. 79 mg/l, *p* = 0.002), with both control and experiment including one vortexing event, it is reasonable to state that centrifugation is by itself a process that induces blood damage. It is difficult to estimate the level of damage induced by experiments published in literature a posteriori, as these often only report the centrifugation speed (rpm) instead of the relative centrifugal forces (rcf), which, without knowledge of the rotor diameter, do not reveal the applied forces (Sutton et al., 1997; Zhao et al., 2006; Quinn et al., 2011). The need to consider such “processing lesions” in addition to the well-known storage lesions was already pointed out by Urbina et al. who noted that the centrifuging performed to extract erythrocyte concentrate from whole blood donations changed RBC shape and MCH (Urbina et al., 2016).

We also observed overall higher values of free hemoglobin for samples centrifuged for 5 min compared to those centrifuged for 10 min. This may be due to an unintended bias toward male subjects in these samples (100% male subjects in samples selected for the 5 min centrifuging steps vs. 25% of male subjects for the samples selected for 10 min). It is known that females have 80% more young RBCs and 85% fewer old RBCs than males (Kameneva et al., 1999), and that old RBCs have a significantly increased mechanical fragility and rigidity compared to young ones (Sutera et al., 1985). Older RBCs are thus more susceptible to damage, which may explain the higher values of plasma free Hb noted in the 5 min, males-only, group. We also observed an increased amount of PS-positive cells in this subject group, which supports this interpretation. The results shown for the other experimental procedures (pressure, pipetting, vortexing) were not affected by this sex bias, since the study used a paired design wherein the blood from each donor was exposed to all tested conditions, including control.

Vortexing and pipetting did not yield any notable changes in the ektacytometry curves, phosphatidylserine expression, or in any of the parameters measured within the CBC (Figures S2, S3, S6, S8). For pipetting, this absence of notable changes may be explained by the relatively low overall damage level that was measured. However, this does not hold true for vortexing, which induced significantly elevated levels of plasma hemoglobin. This may suggest that the noted cell damage resulted from complete cell lysis affecting RBCs uniformly, irrespective of their size and age.

In contrast to the above, centrifuging and elevated pressures were also associated with changes in RBC properties (Figures 3, 4). Centrifuging induced a subtle increase in mean cellular volume, pointing at a destruction of older and smaller RBCs. Also, the maximum deformability decreased with an increasing number of combined resuspension and centrifuging steps (Figure S9D), a behavior that was not observed in the experiments solely involving vortexing (Figure S6D). While the magnitude of the noted changes in mean cellular volume and maximum deformability remained small, these two observations combined are of relevance for both clinical laboratories and researchers: they suggest that even if the remaining cells maintain sufficient membrane integrity not to release significant amounts of intracellular hemoglobin, their mechanical properties may still be altered by repeated centrifugation and resuspension. This finding is also supported by Urbina et al. who observed significant morphological changes, including the appearance of abnormal shapes (echinocytes), after centrifugation (Urbina et al., 2016).

Elevated pressures, on the other hand, led to a decrease of mean RBC volume, a trend toward decreasing MCH and constant MCHC compared to controls. One possible interpretation of the decrease in volume may be the destruction of larger cells. This would be in contrast to previous reports that suggested that mechanical stresses, albeit mostly shear stresses, would affect smaller, older cells first and thus lead to increased MCV (Sakota et al., 2008). Alternatively, this observed cell shrinkage could also point toward a channel-mediated loss of cytosolic content due to the mechanical load (Cahalan et al., 2015). Since we also observed increased levels of plasma free Hb, it could be hypothesized that the weaker cells suffer from complete lysis, while the remainder of the RBC population reacts with cell shrinkage following Ca^2+^ influx (Bogdanova et al., 2013). Otherwise, high pressures have been noted to alter the permeability of the bilayer cell membrane (MacDonald, 1984), such that cytosolic content, including hemoglobin, could be released into the plasma without full cell lysis. Compared to the conditions explored here, those experiments were conducted at extreme pressure levels (250–1,500 bar). Yet, the simultaneous reduction in corpuscular volume and increase in free Hb observed in our pressure experiments could hint at a similar mechanism.

From a clinical perspective, one limitation of this study is the sole investigation of hematologically healthy patients. It might be of interest to characterize whether RBCs with known membrane alterations, such as in patients with sickle cell disease or hereditary spherocytosis, react differently to the same levels of centrifuging, vortexing, and pipetting. Determining whether or not different RBC properties are associated with different reactions to standard procedures would be of relevance for the diagnosis workup, as this could interfere with correct interpretation of results and introduce a bias in the comparison of a given patient's hematological characteristics against standard healthy values. From an experimental perspective, a limitation of this study is the limited applicability of quantitative results to other experimental protocols or setups. This study should thus primarily raise awareness that both preparatory steps as well as secondary factors inherent to an experimental setup should be systematically checked for their influence on the selected study endpoints. Finally, given the small magnitude of the noted changes in CBC parameters, one should also carefully consider potential artifacts introduced by the measurement method. The Advia hematology system uses optical scattering as working principle. We confirmed the trends of increasing MCV after centrifuging and decreasing MCV after exposure to high pressures also in an impedance-based system (Sysmex NX-100, Sysmex K. K., Kobe, Japan), indicating that the noted differences in MCV, although arguably small, were not artefactual. However, manual analysis may be needed for a conclusive answer.

## Conclusion

Within a standard laboratory workflow, there are multiple steps that expose cells to mechanical stress. Here, we investigated the effect of centrifuging, vortexing, pipetting, and pressure on human RBCs. All procedures significantly increased the free hemoglobin in plasma, the measured hemolysis increasing with the vortexing time or the applied pressure. Elevated pressures and centrifugation also altered MCV and MCH. Careful quantification of the influence of these steps as well as of other unwanted secondary effects should be included in experimental protocols and should be checked for in clinical laboratories.

## Author contributions

LW conducted the experiments and performed the data analysis. DdZ supervised the experimental study design and interpretation. OS enabled the measurements of free hemoglobin. AM and JG contributed to the ektacytometry measurements. OS and JG guided the clinical interpretation. BS did the statistical analyses. VK conceived the study and directed the research. All authors wrote the manuscript.

### Conflict of interest statement

The authors declare that the research was conducted in the absence of any commercial or financial relationships that could be construed as a potential conflict of interest. The handling Editor declared a shared affiliation, though no other collaboration, with all the authors.

## References

[B1] Bai G. Bee J. S. Biddlecombe J. G. Chen Q. Leach W. T. (2012). Computational fluid dynamics (CFD) insights into agitation stress methods in biopharmaceutical development. Int. J. Pharm. 423, 264–280. 10.1016/j.ijpharm.2011.11.044 22172288

[B2] Baskurt O. Boynard M. Cokelet G. Connes P. Cooke B. M. Forconi S.. (2009). New guidelines for hemorheological laboratory techniques. Clin. Hemorheol. Microcirc. 42, 75–97. 10.3233/CH-2009-1202 19433882

[B3] Boas F. E. Forman L. Beutler E. (1998). Phosphatidylserine exposure and red cell viability in red cell aging and in hemolytic anemia. Proc. Natl. Acad. Sci. U.S.A. 95, 3077–3081. 10.1073/pnas.95.6.3077 9501218 PMC19697

[B4] Bogdanova A. Makhro A. Wang J. Lipp P. Kaestner L. (2013). Calcium in red blood cells-a perilous balance. Int. J. Mol. Sci. 14, 9848–9872. 10.3390/ijms14059848 23698771 PMC3676817

[B5] Cahalan S. M. Lukacs V. Ranade S. S. Chien S. Bandell M. Patapoutian A. (2015). Piezo1 links mechanical forces to red blood cell volume. Elife 4, 1–12. 10.7554/eLife.07370 26001274 PMC4456639

[B6] Exadactylos A. Geffen A. J. Panagiotaki P. (2003). Population structure of Dover sole *Solea solea*: RAPD and allozyme data indicate divergence in European stocks. Mar. Ecol. Prog. Ser. 246, 253–264. 10.3354/meps246253

[B7] Fairbanks V. F. Ziesmer S. C. O'Brien P. C. (1992). Methods for measuring plasma hemoglobin in micromolar concentration compared. Clin. Chem. 38, 132–140. 1733585

[B8] Ferraro G. A. De Francesco F. Tirino V. Cataldo C. Rossano F. Nicoletti G.. (2011). Effects of a new centrifugation method on adipose cell viability for autologous fat grafting. Aesthetic Plast. Surg. 35, 341–348. 10.1007/s00266-010-9613-8 21069324

[B9] Gunter E. W. Bowman B. A. Caudill S. P. Twite D. B. Adams M. J. Sampson E. J. (1996). Results of an international round robin for serum and whole-blood folate. Clin. Chem. 42, 1689–1694. 8855155

[B10] Heng B. C. Liu H. Ge Z. Cao T. (2007). Mechanical dissociation of human embryonic stem cell colonies by manual scraping after collagenase treatment is much more detrimental to cellular viability than is trypsinization with gentle pipetting. Biotechnol. Appl. Biochem. 47, 33–37. 10.1042/BA20060151 17115976

[B11] Kameneva M. V. Watach M. J. Borovetz H. S. (1999). Gender difference in rheologic properties of blood and risk of cardiovascular diseases. Clin. Hemorheol. Microcirc. 21, 357–363. 10711771

[B12] Katkov I. I. Mazur P. (1998). Influence of centrifugation regimes on motility, yield and cell assications of mouse spermatozoa. J. Androl. 19, 232–241. 10.1002/j.1939-4640.1998.tb01993.x 9570748

[B13] Kay S. S. Bilek A. M. Dee K. C. Gaver D. P. (2004). Pressure gradient, not exposure duration, determines the extent of epithelial cell damage in a model of pulmonary airway reopening. J. Appl. Physiol. 97, 269–276. 10.1152/japplphysiol.01288.2003 15004001

[B14] Kirklin J. K. Naftel D. C. Pagani F. D. Kormos R. L. Stevenson L. W. Blume E. D.. (2015). Seventh INTERMACS annual report: 15,000 patients and counting. J. Hear. Lung Transplant. 34, 1495–1504. 10.1016/j.healun.2015.10.003 26520247

[B15] Korin N. Bransky A. Dinnar U. (2007). Theoretical model and experimental study of red blood cell (RBC) deformation in microchannels. J. Biomech. 40, 2088–2095. 10.1016/j.jbiomech.2006.10.004 17188279

[B16] Kuypers F. A. Lewis R. A. Hua M. Schott M. A. Discher D. Ernst J. D.. (1996). Detection of altered membrane phospholipid asymmetry in subpopulations of human red blood cells using fluorescently labeled annexin V. Blood 87, 1179–1187. 8562945

[B17] Leverett L. Hellums J. Alfrey C. Lynch E. C. (1972). Red blood cell damage by shear stress. Biophys. J. 12, 257–273. 10.1016/S0006-3495(72)86085-5 5016112 PMC1484094

[B18] Lippi G. Guidi G. C. Mattiuzzi C. Plebani M. (2006). Preanalytical variability: the dark side of the moon in laboratory testing. Clin. Chem. Lab. Med. 44, 358–365. 10.1515/CCLM.2006.073 16599826

[B19] Lutz H. U. Bogdanova A. (2013). Mechanisms tagging senescent red blood cells for clearance in healthy humans. Front. Physiol. 4:387. 10.3389/fphys.2013.00387 24399969 PMC3872327

[B20] MacDonald A. G. (1984). The effects of pressure on the molecular structure and physiological functions of cell membranes. Philos. Trans. R. Soc. L. 304, 47–68. 10.1098/rstb.1984.0008 6142479

[B21] Makhro A. Huisjes R. Verhagen L. P. del Manu-Pereira M. Llaudet-Planas E. Petkova-Kirova P.. (2016). Red cell properties after different modes of blood transportation. Front. Physiol. 7:288. 10.3389/fphys.2016.00288 27471472 PMC4945647

[B22] Miao Y. Jiang L. (2007). Transient expression of fluorescent fusion proteins in protoplasts of suspension cultured cells. Nat. Protoc. 2, 2348–2353. 10.1038/nprot.2007.360 17947977

[B23] Paul R. Apel J. Klaus S. Schügner F. Schwindke P. Reul H. (2003). Shear stress related blood damage in laminar Couette flow. Artif. Organs 27, 517–529. 10.1046/j.1525-1594.2003.07103.x 12780506

[B24] Plebani M. (2007). Errors in laboratory medicine and patient safety: the road ahead. Clin. Chem. Lab. Med. 45, 700–707. 10.1515/CCLM.2007.170 17579520

[B25] Quinn D. J. Pivkin I. Wong S. Y. Chiam K.-H. Dao M. Karniadakis G. E.. (2011). Combined simulation and experimental study of large deformation of red blood cells in microfluidic systems. Ann. Biomed. Eng. 39, 1041–1050. 10.1007/s10439-010-0232-y 21240637 PMC3075573

[B26] Rand R. P. (1964). Mechanical properties of the red cell membrane: II. Viscoelastic breakdown of the membrane. Biophys. J. 4, 303–316. 10.1016/S0006-3495(64)86784-9 14197789 PMC1367508

[B27] Rao S. Bálint S. Cossins B. Guallar V. Petrov D. (2009). Raman study of mechanically induced oxygenation state transition of red blood cells using optical tweezers. Biophys. J. 96, 209–216. 10.1529/biophysj.108.139097 18931252 PMC2710025

[B28] Sakota D. Sakamoto R. Sobajima H. Yokoyama N. Waguri S. Ohuchi K.. (2008). Mechanical damage of red blood cells by rotary blood pumps: selective destruction of aged red blood cells and subhemolytic trauma. Artif. Organs 32, 785–791. 10.1111/j.1525-1594.2008.00631.x 18959667

[B29] Shapira Y. Vaturi M. Sagie A. (2009). Hemolysis associated with prosthetic heart valves: a review. Cardiol. Rev. 17, 121–124. 10.1097/CRD.0b013e31819f1a83 19384085

[B30] Sowemimo-Coker S. O. (2002). Red blood cell hemolysis during processing. Transfus. Med. Rev. 16, 46–60. 10.1053/tmrv.2002.29404 11788929

[B31] Sutera S. P. Gardner R. A. Boyland C. W. Carroll G. L. Chang K. C. Marvel J. S.. (1985). Age-related changes in deformability of human erythrocytes. Blood 65, 275–282. 3967082

[B32] Sutton N. Tracey M. C. Johnston I. D. Greenaway R. S. Rampling M. W. (1997). A novel instrument for studying the flow behaviour of erythrocytes through microchannels simulating human blood capillaries. Microvasc. Res. 53, 272–281. 10.1006/mvre.1997.2014 9211405

[B33] Tukey J. W. (1977). Exploratory data analysis, in Addison-Wesley Series in Behavioral Science. Quantitative Methods (Massachusetts: Addison-Wesley Educational Publishers Inc.), 5–23.

[B34] Urbina A. Godoy-Silva R. Hoyos M. Camacho M. (2016). Acute hydrodynamic damage induced by SPLITT fractionation and centrifugation in red blood cells. J. Chromatogr. B Anal. Technol. Biomed. Life Sci. 1020, 53–61. 10.1016/j.jchromb.2016.03.025 27023157

[B35] Zhang Z. Chisti Y. Moo-young M. (1995). Effects of the hydrodynamic environment and shear protectants on survival of erythrocytes in suspension. J. Biotechnol. 43, 33–40. 10.1016/0168-1656(95)00111-8 8573320

[B36] Zhao R. Antaki J. F. Naik T. Bachman T. N. Kameneva M. V. Wu Z. J. (2006). Microscopic investigation of erythrocyte deformation dynamics. Biorheology 43, 747–765. 17148857

