# Supplementary material for: Influence of Standard Laboratory Procedures on Measures of Erythrocyte Damage
Source: Front Physiol. 2017 Sep 29;8:731. doi: 10.3389/fphys.2017.00731 (PMC5632557; doi:10.3389/fphys.2017.00731)
Supplement: Supplementary file 1 [file DataSheet1.docx]

Supplementary Material

Influence of standard laboratory procedures on measures of erythrocyte damage

Lena Wiegmann, Diane de Zélicourt, Oliver Speer, Alissa Muller, Jeroen S. Goede, Burkhardt Seifert, Vartan Kurtcuoglu*

*** Correspondence:** Vartan Kurtcuoglu, vartan.kurtcuoglu@uzh.ch

# Results of Complete Blood Count


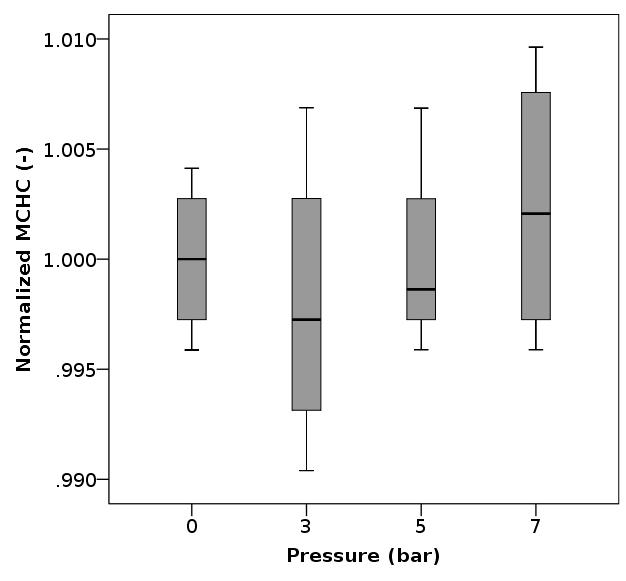


Figure S1: Normalized mean corpuscular hemoglobin concentration as a function of the pressure level after exposure to high pressure. Blood samples were exposed to high pressures 10 times (single exposure duration of 1 s) with a 30 s recovery period between two consecutive exposures. Reported values have been normalized by the mean of the corresponding control measurements. n = 4, 4, 3, 4 for high pressures of 0, 3, 5, and 7 bar, respectively.


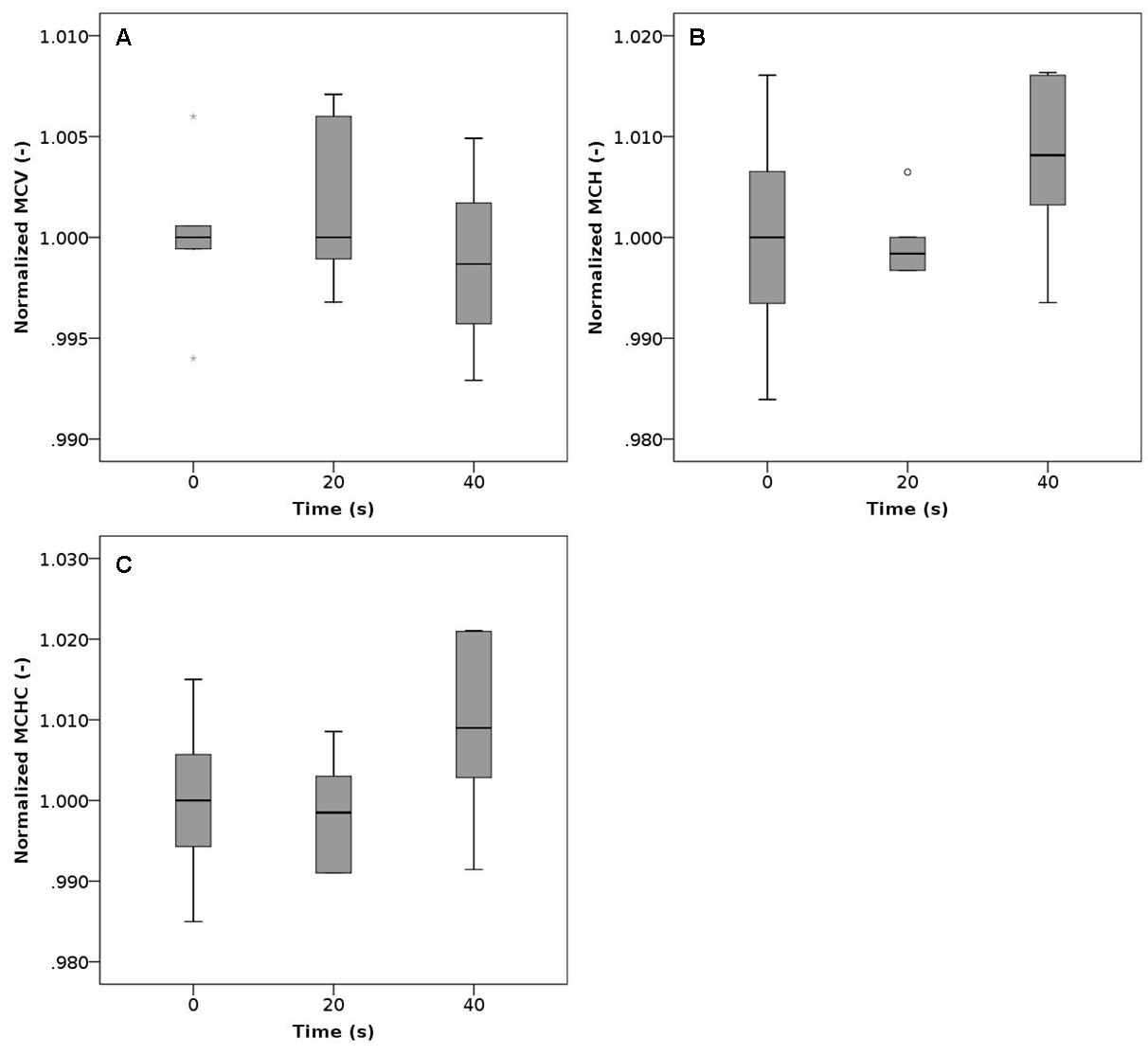


Figure S2: Results of complete blood count after vortexing. (A): Normalized mean cell volume as a function of the vortexing time. (B): Normalized mean corpuscular hemoglobin as a function of the vortexing time. (C): Normalized mean corpuscular hemoglobin concentration as a function of the vortexing time. Reported values have been normalized by the mean of the corresponding control measurements. n per group = 6, for all graphs.


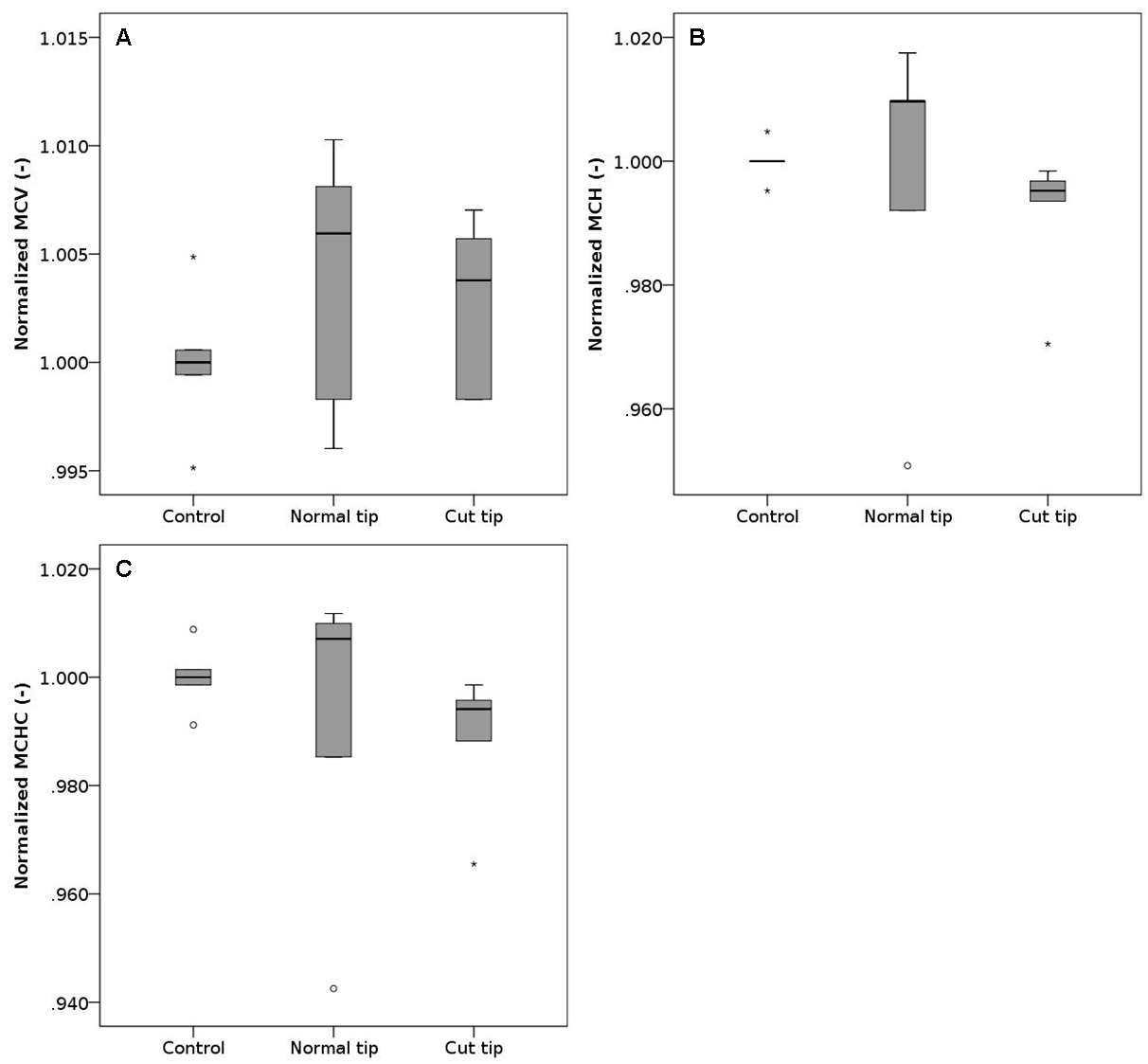


Figure S3: Results of complete blood count after 10 times pipetting using normal or cut tips. (A): Normalized mean cell volume. (B): Normalized mean corpuscular hemoglobin. (C): Normalized mean corpuscular hemoglobin concentration. Reported values have been normalized by the mean of the corresponding control measurements. n per group = 5, for all graphs.

# Results of ektacytometry measurements


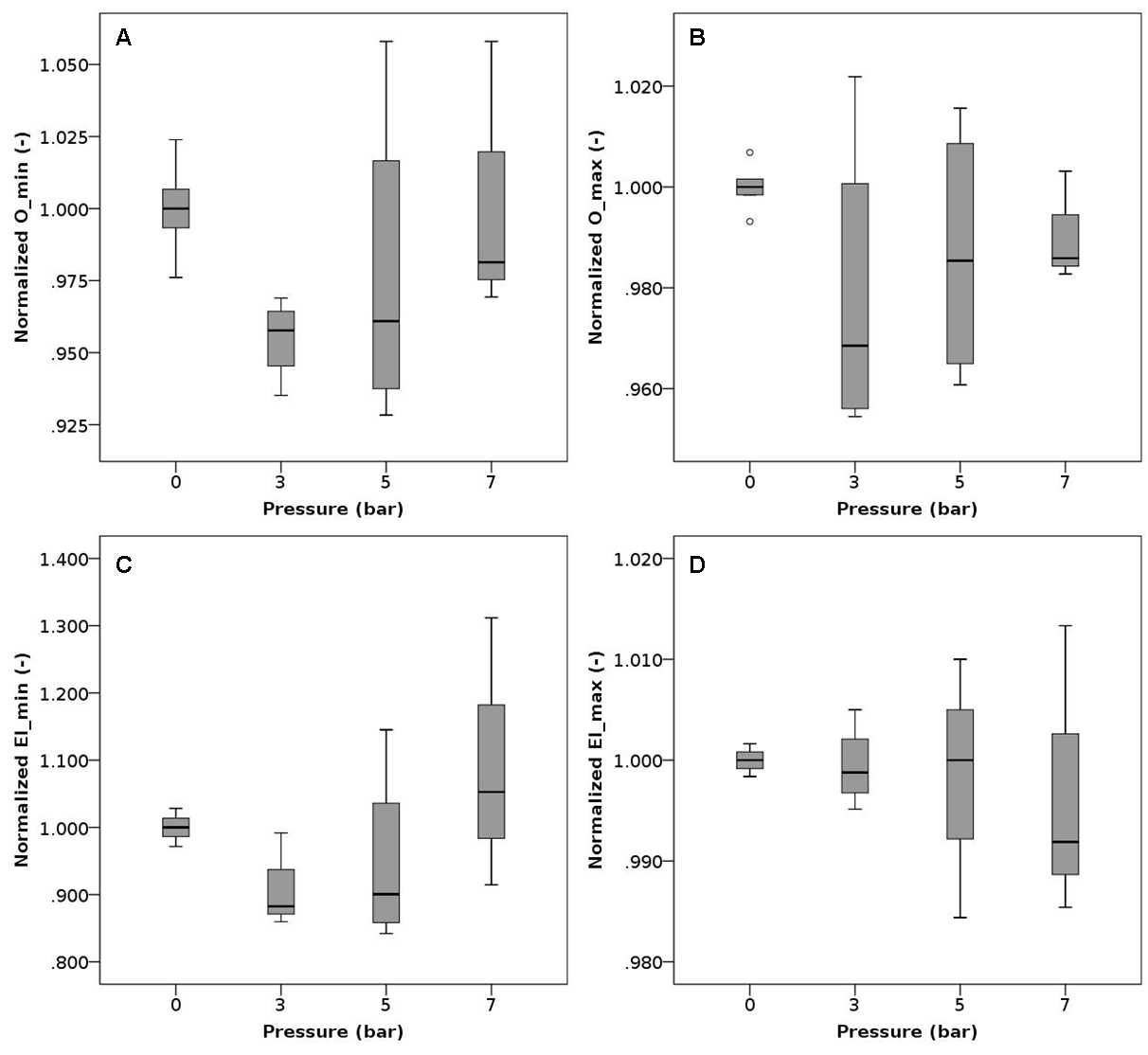


Figure S4: Results of ektacytometry measurements after exposure to high pressure. Blood samples were exposed to high pressures 10 times (single exposure duration of 1 s) with a 30 s recovery period between two consecutive exposures. (A): Normalized O_min_ as a function of the pressure level. O_min_ is the osmolality at which the minimal elongation index EI_min_ is measured. (B): Normalized O_max_ as a function of the pressure level. O_max_ is the osmolality at which the maximal elongation index EI_max_ is measured (C): Normalized minimal elongation index EI_min_ as a function of the pressure level. (D): Normalized maximal elongation index EI_max_ as a function of the pressure level. Reported values have been normalized by the mean of the corresponding control measurements. n = 5, 4, 4, 3 for high pressures of 0, 3, 5, 7 bar respectively, for all graphs.


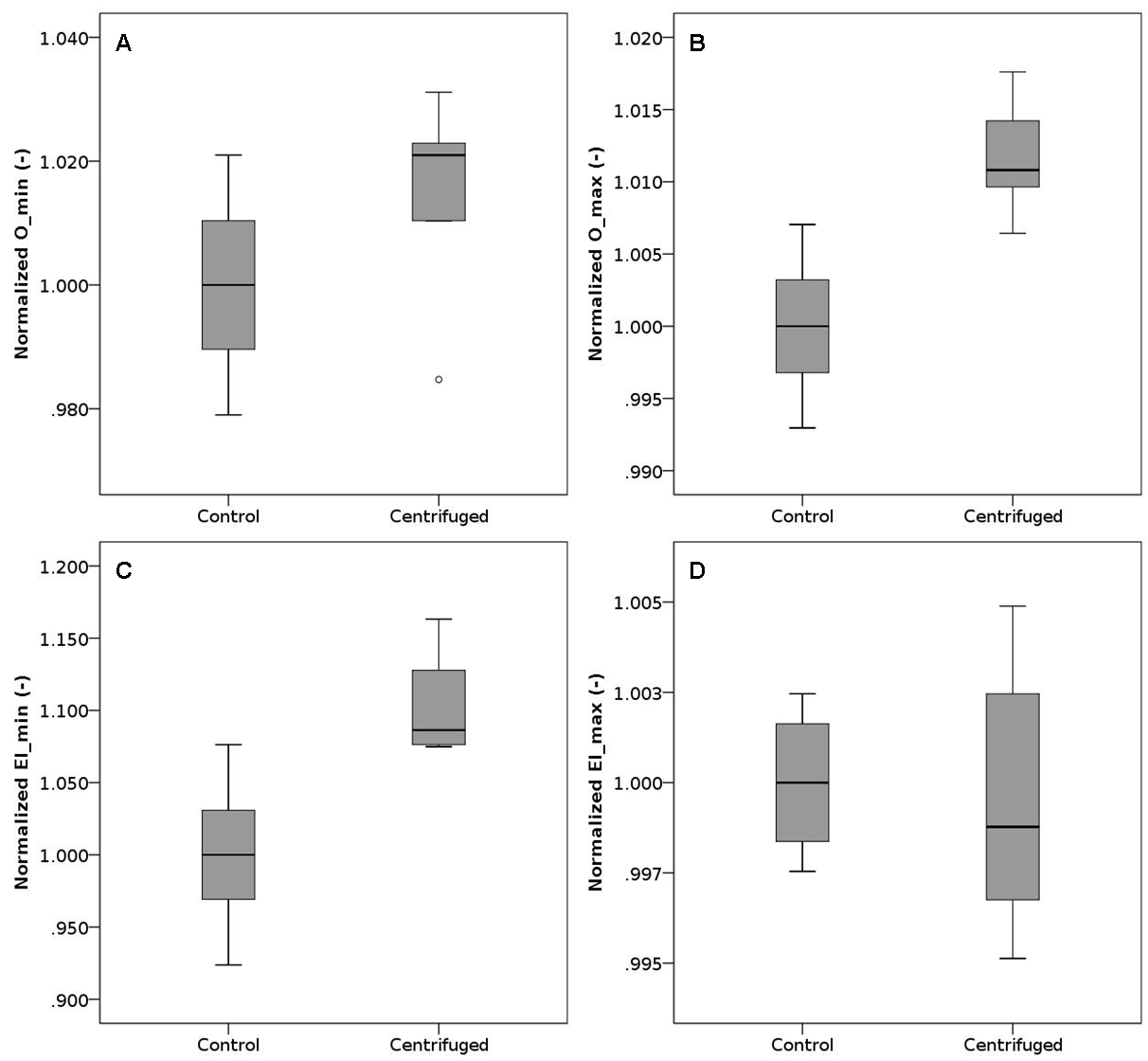


Figure S5: Results of ektacytometry measurements after centrifugation at 900g for 5 minutes. (A): Normalized O_min_. O_min_ is the osmolality at which the minimal elongation index EI_min_ is measured. (B): Normalized O_max_. O_max_ is the osmolality at which the maximal elongation index EI_max_ is measured (C): Normalized minimal elongation index EI_min_. (D): Normalized maximal elongation index EI_max_. Reported values have been normalized by the mean of the corresponding control measurements. n per group = 6, for all graphs.


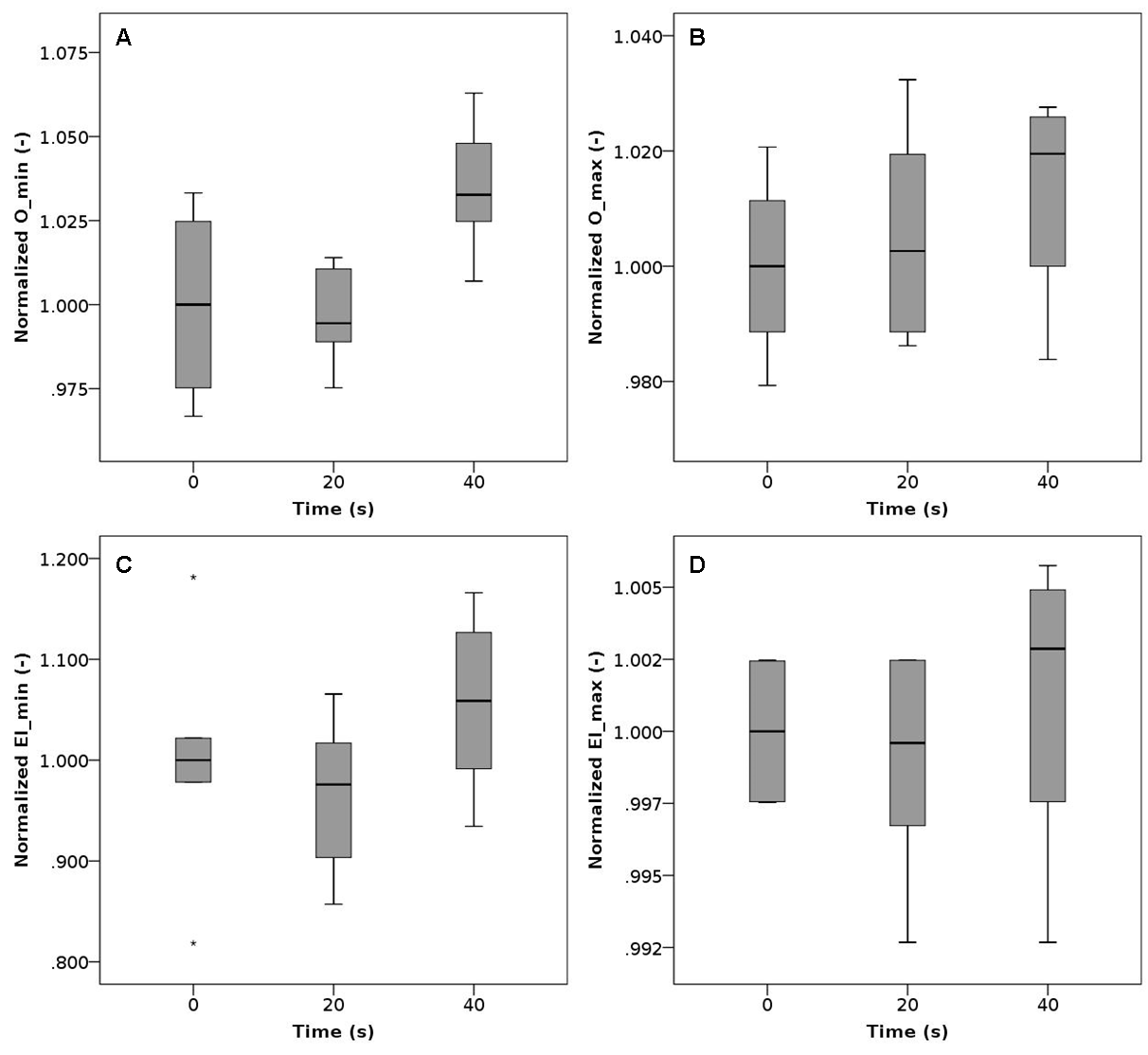


Figure S6: Results of ektacytometry measurements after vortexing. (A): Normalized O_min_ as a function of the vortexing time. O_min_ is the osmolality at which the minimal elongation index EI_min_ is measured. (B): Normalized O_max_ as a function of the vortexing time. O_max_ is the osmolality at which the maximal elongation index EI_max_ is measured (C): Normalized minimal elongation index EI_min_ as a function of the vortexing time. (D): Normalized maximal elongation index EI_max_ as a function of the vortexing time. Reported values have been normalized by the mean of the corresponding control measurements. n = 5, 6, 6 for vortexing of 0s, 20s and 40s, respectively, for all graphs.


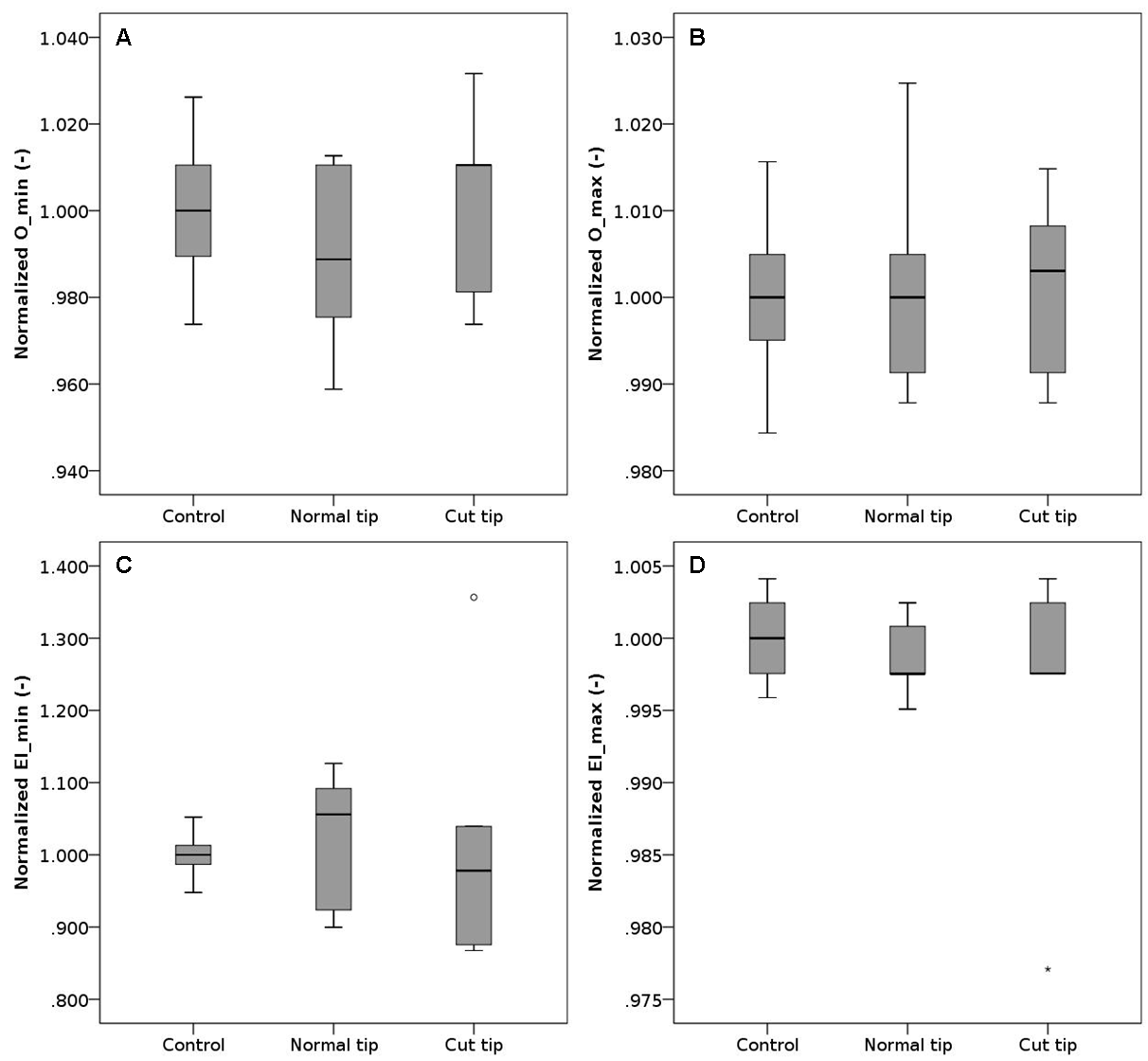


Figure S7: Results of ektacytometry measurements after pipetting with normal versus cut tips. (A): Normalized O_min_ after pipetting with normal vs. cut tips. O_min_ is the osmolality at which the minimal elongation index EI_min_ is measured. (B): Normalized O_max_ after pipetting with normal vs. cut tips. O_max_ is the osmolality at which the maximal elongation index EI_max_ is measured. (C): Normalized minimal elongation index EI_min_ after pipetting with normal vs. cut tips. (D): Normalized maximal elongation index EI_max_ after pipetting with normal vs. cut tips. Reported values have been normalized by the mean of the corresponding control measurements. n per group = 5, for all graphs

# Results of phosphatidylserine measurements


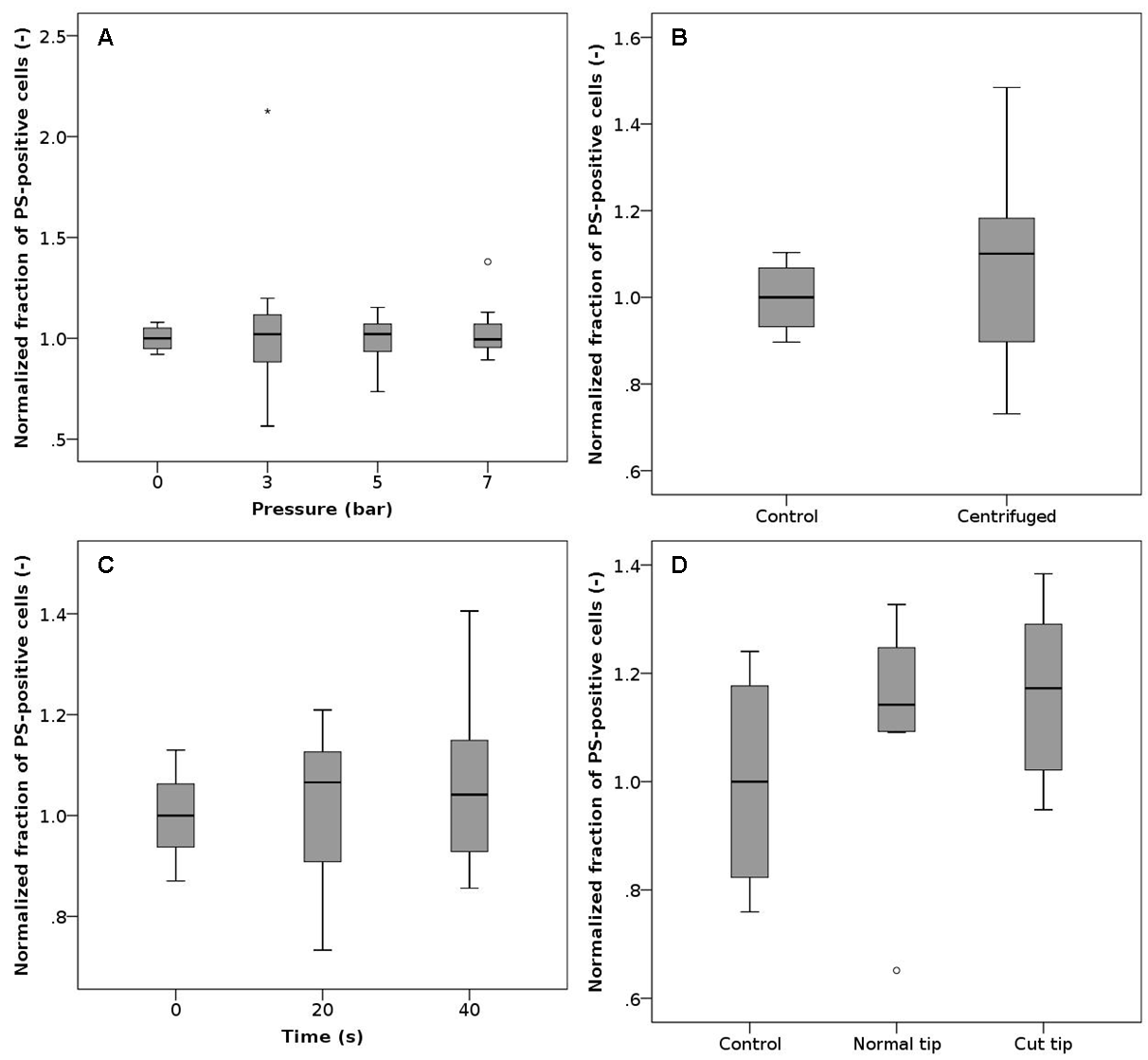


Figure S8: Percentage of PS-positive red blood cells measured by flow cytometry. (A): Normalized fraction of Phosphatidylserine-positive red blood cells as a function of the applied pressure level (exposure time 1s, 10 consecutive times with 30s recovery). n per group = 8. (B): Normalized fraction of Phosphatidylserine-positive red blood cells after centrifugation at 900g for 5 minutes. n = 7, 8 for control and centrifuged samples, respectively. (C): Normalized fraction of Phosphatidylserine-positive red blood cells as a function of the vortexing time. n = 7, 8, 8 for vortexing during 0s, 20s and 40s, respectively. (D): Normalized fraction of Phosphatidylserine-positive red blood cells after pipetting with normal vs. cut tips. n per group = 8. Reported values have been normalized by the mean of the corresponding control measurements.

# Results of multiple centrifugation and resuspension steps


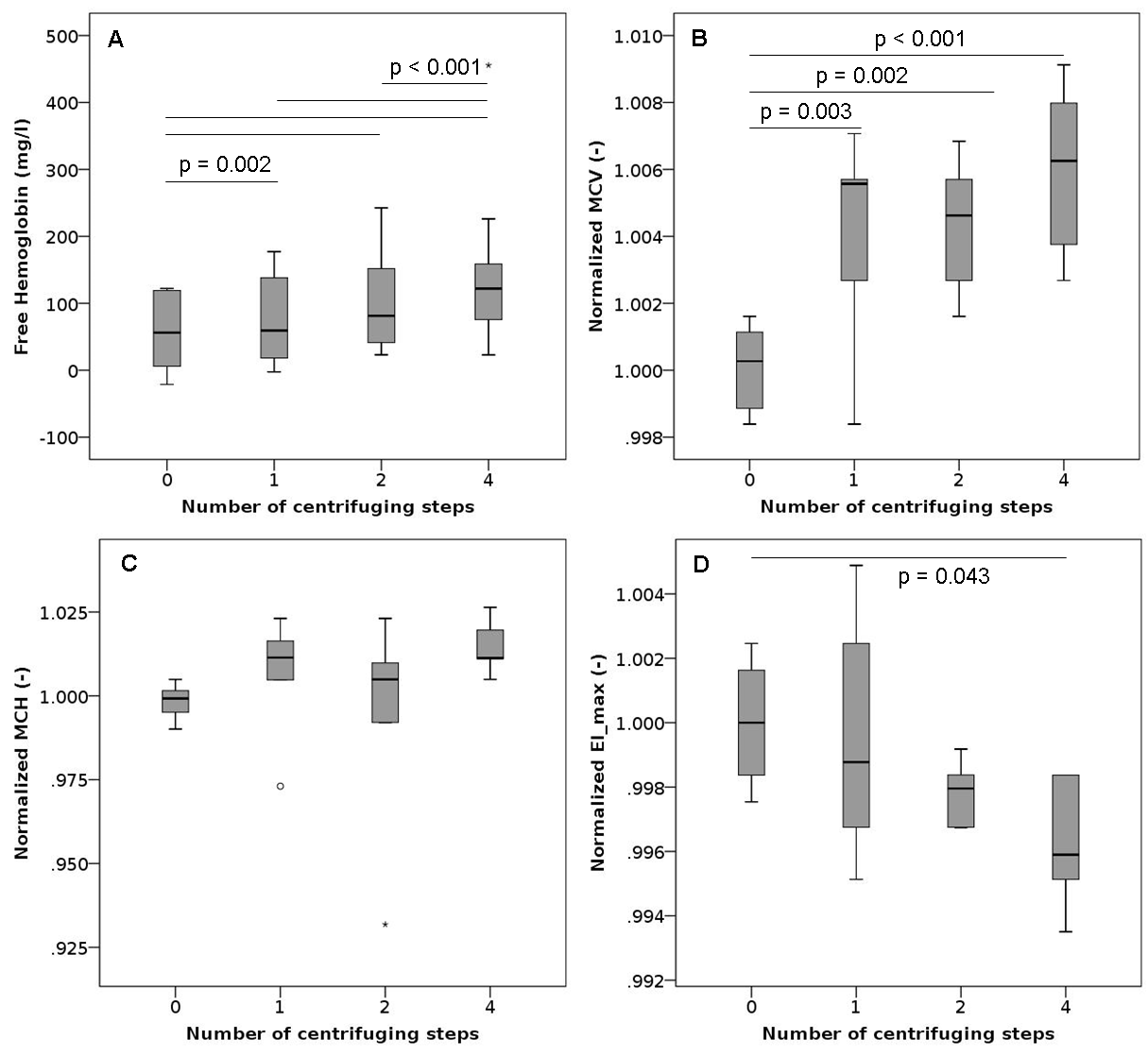


Figure S9: Free hemoglobin, MCV, MCH and EI_max_ after repeated centrifugation and resuspension. Samples were centrifuged at 900 g and vortexed for 2s between two consecutive centrifugation steps. (A): Free hemoglobin as a function of the number of centrifugation and resuspension steps. time per step = 5 or 10 min, n per group = 12. (B) Normalized mean cell volume as a function of the number of centrifugation and resuspension steps. Reported values have been normalized by the mean of the corresponding control measurements. time per step = 5 min. n per group = 6. (C) Normalized mean corpuscular hemoglobin as a function of the number of centrifugation and resuspension steps. Reported values have been normalized by the mean of the corresponding control measurements. time per step = 5 min, n per group = 6. (D): Normalized maximal elongation index EI_max_ as a function of the number of centrifuging and resuspension steps. Reported values have been normalized by the mean of the corresponding control measurements. time per step = 5 min, n per group = 6.
